# Incidence of diabetes after SARS-CoV-2 infection in England and the implications of COVID-19 vaccination: a retrospective cohort study of 16 million people

**DOI:** 10.1016/S2213-8587(24)00159-1

**Published:** 2024-08-01

**Authors:** Kurt Taylor, Sophie Eastwood, Venexia Walker, Genevieve Cezard, Rochelle Knight, Marwa Al Arab, Yinghui Wei, Elsie M F Horne, Lucy Teece, Harriet Forbes, Alex Walker, Louis Fisher, Jon Massey, Lisa E M Hopcroft, Tom Palmer, Jose Cuitun Coronado, Samantha Ip, Simon Davy, Iain Dillingham, Caroline Morton, Felix Greaves, John Macleod, Ben Goldacre, Angela Wood, Nishi Chaturvedi, Jonathan A C Sterne, Rachel Denholm

**Affiliations:** Population Health Sciences, University of Bristol, Bristol, UK; MRC Integrative Epidemiology Unit, University of Bristol, Bristol, UK; MRC Unit for Lifelong Health and Ageing, University College London, London, UK; Population Health Sciences, University of Bristol, Bristol, UK; Integrative Epidemiology Unit, University of Bristol, Bristol, UK; Department of Surgery, University of Pennsylvania Perelman School of Medicine, Philadelphia, PA, USA; The Bennett Institute for Applied Data Science, Nuffield Department of Primary Care Health Sciences, University of Oxford, Oxford, UK; British Heart Foundation Cardiovascular Epidemiology Unit, Department of Public Health and Primary Care; Victor Phillip Dahdaleh Heart and Lung Research Institute; Population Health Sciences, University of Bristol, Bristol, UK; MRC Integrative Epidemiology Unit, University of Bristol, Bristol, UK; NIHR Bristol Biomedical Research Centre, Bristol, UK; The National Institute for Health and Care Research Applied Research Collaboration West (NIHR ARC West) at University Hospitals Bristol and Weston, Bristol, UK; Population Health Sciences, University of Bristol, Bristol, UK; Centre for Mathematical Sciences, School of Engineering, Computing and Mathematics, University of Plymouth, Plymouth, UK; Population Health Sciences, University of Bristol, Bristol, UK; NIHR Bristol Biomedical Research Centre, Bristol, UK; Department of Population Health Sciences, University of Leicester, Leicester, UK; Faculty of Epidemiology and Population Health, London School of Hygiene & Tropical Medicine, London, UK; The Bennett Institute for Applied Data Science, Nuffield Department of Primary Care Health Sciences, University of Oxford, Oxford, UK; The Bennett Institute for Applied Data Science, Nuffield Department of Primary Care Health Sciences, University of Oxford, Oxford, UK; The Bennett Institute for Applied Data Science, Nuffield Department of Primary Care Health Sciences, University of Oxford, Oxford, UK; Population Health Sciences, University of Bristol, Bristol, UK; MRC Integrative Epidemiology Unit, University of Bristol, Bristol, UK; Population Health Sciences, University of Bristol, Bristol, UK; British Heart Foundation Cardiovascular Epidemiology Unit, Department of Public Health and Primary Care; Centre for Cancer Genetic Epidemiology, Department of Public Health and Primary Care, University of Cambridge, Cambridge, UK; The Bennett Institute for Applied Data Science, Nuffield Department of Primary Care Health Sciences, University of Oxford, Oxford, UK; The Bennett Institute for Applied Data Science, Nuffield Department of Primary Care Health Sciences, University of Oxford, Oxford, UK; The Bennett Institute for Applied Data Science, Nuffield Department of Primary Care Health Sciences, University of Oxford, Oxford, UK; National Institute for Health and Care Excellence, Manchester, UK; Department of Primary Care and Public Health, Imperial College London, London, UK; Population Health Sciences, University of Bristol, Bristol, UK; NIHR Bristol Biomedical Research Centre, Bristol, UK; The National Institute for Health and Care Research Applied Research Collaboration West (NIHR ARC West) at University Hospitals Bristol and Weston, Bristol, UK; The Bennett Institute for Applied Data Science, Nuffield Department of Primary Care Health Sciences, University of Oxford, Oxford, UK; British Heart Foundation Cardiovascular Epidemiology Unit, Department of Public Health and Primary Care; Victor Phillip Dahdaleh Heart and Lung Research Institute; British Heart Foundation Centre of Research Excellence, University of Cambridge, Cambridge, UK; National Institute for Health and Care Research Blood and Transplant Research Unit in Donor Health and Behaviour, University of Cambridge, Cambridge, UK; Cambridge Centre of Artificial Intelligence in Medicine, University of Cambridge, Cambridge, UK; Health Data Research UK Cambridge, Wellcome Genome Campus and University of Cambridge, Cambridge, UK; MRC Unit for Lifelong Health and Ageing, University College London, London, UK; Population Health Sciences, University of Bristol, Bristol, UK; NIHR Bristol Biomedical Research Centre, Bristol, UK; Health Data Research UK South-West, Bristol, UK; Population Health Sciences, University of Bristol, Bristol, UK; NIHR Bristol Biomedical Research Centre, Bristol, UK; THealth Data Research UK South-West, Bristol, UK

## Abstract

**Background:**

Some studies have shown that the incidence of type 2 diabetes increases after a diagnosis of COVID-19, although the evidence is not conclusive. However, the effects of the COVID-19 vaccine on this association, or the effect on other diabetes subtypes, are not clear. We aimed to investigate the association between COVID-19 and incidence of type 2, type 1, gestational and non-specific diabetes, and the effect of COVID- 19 vaccination, up to 52 weeks after diagnosis.

**Methods:**

In this retrospective cohort study, we investigated the diagnoses of incident diabetes following COVID-19 diagnosis in England in a pre-vaccination, vaccinated, and unvaccinated cohort using linked electronic health records. People alive and aged between 18 years and 110 years, registered with a general practitioner for at least 6 months before baseline, and with available data for sex, region, and area deprivation were included. Those with a previous COVID-19 diagnosis were excluded. We estimated adjusted hazard ratios (aHRs) comparing diabetes incidence after COVID-19 diagnosis with diabetes incidence before or in the absence of COVID-19 up to 102 weeks after diagnosis. Results were stratified by COVID-19 severity (categorised as hospitalised or non-hospitalised) and diabetes type.

**Findings:**

16 669 943 people were included in the pre-vaccination cohort (Jan 1, 2020–Dec 14, 2021), 12 279 669 in the vaccinated cohort, and 3 076 953 in the unvaccinated cohort (both June 1–Dec 14, 2021). In the pre-vaccination cohort, aHRs for the incidence of type 2 diabetes after COVID-19 (compared with before or in the absence of diagnosis) declined from 4·30 (95% CI 4·06–4·55) in weeks 1–4 to 1·24 (1·14–1.35) in weeks 53–102. aHRs were higher in unvaccinated people (8·76 [7·49–10·25]) than in vaccinated people (1·66 [1·50–1·84]) in weeks 1–4 and in patients hospitalised with COVID-19 (pre-vaccination cohort 28·3 [26·2–30·5]) in weeks 1–4 declining to 2·04 [1·72–2·42] in weeks 53–102) than in those who were not hospitalised (1·95 [1·78–2·13] in weeks 1–4 declining to 1·11 [1·01–1·22] in weeks 53–102). Type 2 diabetes persisted for 4 months after COVID-19 in around 60% of those diagnosed. Patterns were similar for type 1 diabetes, although excess incidence did not persist beyond 1 year after a COVID-19 diagnosis.

**Interpretation:**

Elevated incidence of type 2 diabetes after COVID-19 is greater, and persists for longer, in people who were hospitalised with COVID-19 than in those who were not, and is markedly less apparent in people who have been vaccinated against COVID-19. Testing for type 2 diabetes after severe COVID-19 and the promotion of vaccination are important tools in addressing this public health problem.

**Funding:**

UK National Institute for Health and Care Research, UK Research and Innovation (UKRI) Medical Research Council, UKRI Engineering and Physical Sciences Research Council, Health Data Research UK, Diabetes UK, British Heart Foundation, and the Stroke Association.

## Introduction

At least 700 million people worldwide have been infected with SARS-CoV-2.^1,2^ Reports of an excess risk of diabetes after COVID-19^3–9^ therefore have alarming public health implications. A 30–50% excess incidence of type 2 diabetes after SARS-CoV-2 infection has been reported.^3–6,9,10^ by contrast, the only three studies that were sufficiently powered for such analyses found no association between type 1 diabetes and SARS-CoV-2.^11–13^ A key question is whether excess diabetes after COVID-19 is driven by short-term causes of hyperglycaemia (stress-induced or steroid-induced) or is a durable consequence of infection. Yet most studies examined diabetes incidence only at a fixed timepoint after COVID-19,^3,4,7,8^ and those that stratified by time period post-infection do not concur.^3–5,7,9^ COVID-19 severity, and therefore COVID-19 vaccination, is likely to be a key determinant of downstream sequelae;^4–9,11,12^ however, only one study, which used data from the US Department of Veterans Affairs and consisted pre-dominantly of White men, examined the effect of COVID-19 vaccination on the incidence of diabetes after COVID-19.^9^

In this study, we quantified associations between COVID-19 and incident diabetes diagnoses using a UK database of linked COVID-19 testing data and primary and secondary care records. We investigated how associations varied: at different timepoints up to 2 years after a COVID-19 diagnosis, by diabetes type, according to COVID-19 vaccination availability and vaccination status, by COVID-19 severity, and within population subgroups.

## Methods

### Study design and participants

In this observational cohort study, we included data from all people aged 18 years or older, registered with a primary care general practice using TPP software in England. Linked data were accessed and analysed securely within the OpenSAFELY platform, which includes individual-level, primary care electronic health records from 24 million people (covering around 40% of the population of England), linked to the Second Generation Surveillance System (SGSS) for pillar 1 and pillar 2 SARS-COV-2 infection laboratory testing data, National Health Service (NHS) hospital admissions (Secondary Uses Services data), and the Office of National Statistics death registry, which includes causes of death.^14^ COVID-19 vaccination records (from the National Immunisation Management System) are available within TPP primary care data.

The UK’s COVID-19 vaccine roll-out started on Dec 8, 2020, with timing of eligibility determined by the Joint Committee on Vaccination and Immunisation (JCVI) on the basis of age, clinical vulnerability, and health and social care occupation.^15^ All adults in England were eligible to receive a first vaccination by June 18, 2021, and a second vaccination by Aug 31, 2021.^16^

We defined three cohorts, summarised in the appendix (p 5). In the pre-vaccination cohort, follow-up started on Jan 1, 2020 (baseline) and ended on the earliest of Dec 14, 2021 (study end date, after which the omicron variant [B.1.1.529] became dominant in England),^19^ the recorded outcome event date, or the date of death. Exposure was defined as a COVID-19 diagnosis (see Procedures) between baseline and the earliest of eligibility for COVID-19 vaccination (based on age and clinical vulnerability) or date of first vaccination; this exposure period was before the delta variant (B.1.617.2) became dominant in England. The other two cohorts were followed during the period when the delta variant was dominant in England: between June 1, 2021 (baseline) and Dec 14, 2021. Follow-up in the vaccinated cohort started at the later of baseline or 2 weeks after a second COVID-19 vaccination and ended at the earliest of the study end date, recorded outcome event date, or date of death. The unvaccinated cohort had not received a COVID-19 vaccine by 12 weeks after they became eligible for vaccination. Follow-up in this cohort started at the later of baseline or 12 weeks after eligibility for vaccination and ended at the earliest of the study end date, recorded outcome event date, date of death, or date of first vaccination.

People eligible for each cohort had to have been registered for at least 6 months with an English general practitioner using TPP software before the cohort baseline; be alive and aged between 18 years and 110 years at baseline; and have available data for sex, region, and area deprivation. People with a history of SARS-CoV-2 infection or COVID-19 diagnosis before the cohort baseline were excluded. There were no exclusion criteria relating to comorbidity status. In the vaccinated cohort, people who received a COVID-19 vaccination before Dec 8, 2020, had a record of receiving a second dose before (ie, an administrative error) or less than 3 weeks after their first dose, or who had received more than one type of vaccine (eg, first dose AstraZeneca [ChADOx1] vaccine and second dose Pfizer [BNT162b2] vaccine) before May 7, 2021, were excluded. In the unvaccinated cohort, people who could not be assigned to a vaccination priority group as defined by the JCVI were excluded.

People were excluded if they had ever been diagnosed with any diabetes phenotype (see Outcomes) before the cohort start date. For gestational diabetes, the study population was restricted to women.

The protocol and publicly available code lists and analysis code are available online. This study was approved by the Health Research Authority (REC reference 22/PR/0095) and by the University of Bristol’s Faculty of Health Sciences Ethics Committee (reference 117269).

### Procedures

The date of COVID-19 diagnosis was defined as the earliest of the date of a positive SARS-CoV-2 PCR or antigen test in the SGSS system, a confirmed COVID-19 diagnosis in primary or secondary care, or death with SARS-CoV-2 infection listed as primary or underlying cause. People hospitalised with COVID-19 as the primary diagnosis within 28 days of first diagnosis were defined as hospitalised with COVID-19, all others were defined as having COVID-19 without hospitalisation (non-hospitalised).

Primary and secondary care records up to the cohort start date were used to define age, sex, ethnicity, socioeconomic deprivation (using an area-based index of multiple deprivation quintiles), smoking status, and region. We defined the following additional potential confounding variables: health service use (measured by primary care consultation rate), previous health events (ie, myocardial infarction, stroke, arterial embolism and venous thromboembolic events, and gestational diabetes diagnosis), comorbidities (ie, heart failure, angina, dementia, liver disease, chronic kidney disease, cancer, hypertension, depression, chronic obstructive pulmonary disease, and prediabetes), BMI, health-care work status, and residency in a care home (appendix p 6).

### Outcomes

Diabetes phenotypes were defined using primary care and hospital admission data (appendix p 8). An updated, clinician-verified SNOMED-CT and ICD-10 diabetes diagnostic adjudication algorithm^17,18^ was used to define incident diagnosis of type 1 and type 2 diabetes, gestational diabetes, other or non-specific diabetes, and diabetes unlikely (appendix p 13). In the pre-vaccination cohort, to differentiate type 2 diabetes from temporary steroid-induced or stress-induced hyperglycaemia, a secondary outcome of persistent type 2 diabetes was defined as type 2 diabetes with continued treatment (two or more prescriptions of glucose-lowering medication) or elevated HbA_1c_ concentrations (≥47·5 mmol) 4 months after diagnosis.

### Statistical analyses

We described baseline demographic and clinical characteristics of each cohort and calculated the number of events per outcome, person-years of follow-up, and incidence rates (per 100 000 person-years) of events before or in the absence of and after COVID-19, for the whole study population, those defined as hospitalised with COVID-19, and those defined as having COVID-19 without hospitalisation. We calculated age–sex-standardised incidence rates before and after COVID-19, standardised to the pre-vaccination cohort population.

For each diabetes phenotype, we analysed time to first event. Cox models were fitted with calendar time scale using the cohort-specific baseline as the origin (time zero). We estimated hazard ratios (HRs) comparing follow-up after COVID-19 with follow-up before or in the absence of COVID-19, splitting follow-up time into the time periods 1–4 weeks after COVID-19 and 5–28 weeks after COVID-19 for all cohorts and, additionally for the pre-vaccination cohort, 29–52 weeks after COVID-19 and 53–102 weeks after COVID-19. All models were stratified by region to account for between-region variation. For each outcome and cohort, we estimated age and sex and maximally (including all potential confounders) adjusted HRs. We conducted subgroup analyses according to whether people had been hospitalised with COVID-19 within 28 days of their first COVID-19 diagnosis. For type 2 diabetes only, we conducted additional sub-group analyses by age group (18–39, 40–59, 60–79, or 80–110 years), sex, ethnicity, history of prediabetes, and obesity. We derived absolute excess risks of type 2 diabetes after COVID-19, weighted by the proportions of individuals in age and sex strata in the pre-vaccination cohort. Further details of the statistical analyses are provided in the appendix (p 4).

We conducted sensitivity analyses for type 2 diabetes outcomes only. Our analyses implicitly assumed that, when diabetes and COVID-19 diagnoses were made on the same date, the diabetes diagnosis was after the COVID-19 diagnosis. To explore the influence of simultaneous reporting of the exposure and outcome, we repeated the main analysis separating events on day 0 (day of COVID-19 diagnosis) from the rest of weeks 1–4.

Data management and analyses were conducted in Python version 3.8.10 and R version 4.0.2. For disclosure control, all counts have been rounded up to the nearest 6 then subtracted by 3 to obscure counts.

### Role of the funding source

The funder of the study had no role in study design, data collection, data analysis, data interpretation, or writing of the report.

## Results

Of the 33 404 025 individuals in OpenSAFELY-TPP who were eligible for the pre-vaccination cohort, 16 699 943 met the inclusion criteria for the study. Amongst the 33 023 366 participants alive at the start of the delta era, 12 279 699 were included in the vaccinated cohort and 3 076 951 were included in the unvaccinated cohort. Participant selection into each cohort is shown in the appendix (p 14). The follow-up periods were Jan 1, 2020–Dec 14, 2021 for the pre-vaccination cohort and June 1–Dec 14, 2021 for the vaccinated and unvaccinated cohorts.

Among 16 699 943 people in the pre-vaccination cohort, 916 802 (5·5%) were diagnosed with COVID-19 during the study period (table 1); corresponding numbers were 774 475 (6·3%) of 12 279 669 people in the vaccinated cohort and 153 941 (5·0%) of 3 076 951 people in the unvaccinated cohort. 18 669 (0.1%) people in the pre-vaccination cohort, 2787 (0·2%) in the vaccinated cohort, and 981 (0·3%) in the unvaccinated cohort died within 28 days of their initial COVID-19 diagnosis (appendix p 9). Compared with the vaccinated cohort, people who remained unvaccinated were younger (61·1% *vs* 28·7% aged ≤40 years); more likely to be men (58·2% *vs* 47·3%); more likely to be from south Asian (9·6% *vs* 5·1%), Black (5·2% *vs* 1·5%), and other (table 1; 5·9% and 1·7%) ethnic backgrounds than a White ethnic background; and more likely to be from the most deprived background (29·5% *vs* 15·7%). People in the unvaccinated cohort had fewer comorbidities than those in the vaccinated cohort (appendix p 9).

The median follow-up times were 714 (IQR 714–714) days in the pre-vaccination cohort, 190 (147–197) days in the vaccinated cohort, and 126 (106–172) days in the unvaccinated cohort. Almost all people in the pre-vaccinated cohort were followed for the duration of the study period, therefore the median and the bounds of the IQR are the same. 145 533 people were diagnosed with incident type 2 diabetes in the pre-vaccination cohort, 34 365 in the vaccinated cohort, and 2781 in the unvaccinated cohort (table 2). Corresponding numbers of incident type 1 diabetes events were 16 047 in the pre-vaccination cohort, 2619 in the vaccinated cohort, and 747 in the unvaccinated cohort. The incidence of both type 2 and type 1 diabetes before or in the absence of a COVID-19 diagnosis was higher in the older, vaccinated cohort (median age 51 [IQR 37–65] years) than in the younger, unvaccinated cohort (35 [27–45] years); for example, the incidence rate for type 2 diabetes was 608 per 100 000 person-years in the vaccinated cohort compared with 222 per 100 000 person-years in the unvaccinated cohort (table 2). Age–sex-standardised incidence rates after COVID-19 diagnosis for diabetes phenotypes were consistently higher in the unvaccinated cohort than in the pre-vaccination and vaccinated cohorts (appendix p 10). In all cohorts, the incidence of both type 2 and type 1 diabetes was substantially greater in those hospitalised with COVID-19 than those who did not have COVID-19 or those who were not hospitalised with COVID-19. Among people in the pre-vaccination cohort with follow-up data for at least 4 months after diagnosis, 145 323 diagnoses of incident type 2 diabetes were recorded, of which 89 517 (61·6%) were defined as persistent type 2 diabetes. Of the 6114 people with a type 2 diabetes diagnosis after COVID-19 and at least 4 months of follow-up data, 3486 (57·0%) of cases were defined as persistent type 2 diabetes. The proportion of persistent type 2 diabetes was slightly higher after hospitalisation with COVID-19 (837 [60·5%] of 1383) than after COVID-19 without hospitalisation (2649 [56·0%] of 4731; table 2).

**Figure 1:**
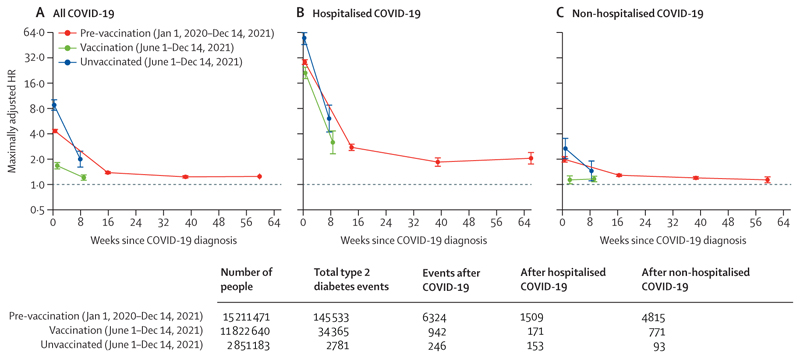
Maximally adjusted HRs comparing the incidence of type 2 diabetes events after COVID-19 with the incidence before or in the absence of COVID-19 in all cohorts Points are plotted at the median time of the outcome event within each follow-up period in each cohort. Error bars are 95% CI. HR=hazard ratio.

In the pre-vaccination cohort, maximally adjusted HRs (aHRs) comparing the incidence of type 2 diabetes after COVID-19 with the incidence before or in the absence of COVID-19 were attenuated compared with age-adjusted, sex-adjusted, and region-adjusted HRs (table 3). However, maximally adjusted and age–sex adjusted aHRs were similar in the vaccinated and unvaccinated cohorts. In each cohort, the incidence of type 2 diabetes was increased during the first 4 weeks after COVID-19 diagnosis; this increase was greater in the unvaccinated cohort (aHR 8·76 [95% CI 7·49–10·25]) than in the vaccinated cohort (1·66 [1·50–1·84]; figure 1, table 3). In the pre-vaccination cohort, the incidence of type 2 diabetes remained elevated 53–102 weeks after diagnosis (aHR 1·24 [1·14–1·35]). The increase in type 2 diabetes incidence 5–28 weeks after COVID-19 was less marked in the pre-vaccination (aHR 1·38 [1·32–1·43]) and vaccinated (1·20 [1·10–1·31]) cohorts than in the unvaccinated cohort (1·99 [1·58–2·50). In the pre-vaccination cohort, the aHRs for persistent type 2 diabetes were lower than for all type 2 diabetes during weeks 1–4, similar during weeks 5–28 and weeks 29–52, and higher during weeks 53–102 (table 3, appendix p16).

The aHRs comparing the incidence of type 2 diabetes after COVID-19 with the incidence before or in the absence of COVID-19 were higher among those hospitalised with COVID-19, than for those who were not, in each cohort (eg, 28·3 [26·2–30·5] *vs* 1·95 [1·78–2·13] in weeks 1–4 in the pre-vaccination cohort; table 3, figure 1). The incidence of type 2 diabetes in those hospitalised with COVID-19 remained elevated beyond 4 weeks after COVID-19 diagnosis in each cohort. In the pre-vaccination cohort, the aHR for incident type 2 diabetes after COVID-19 without hospitalisation was 1·11 (1·01–2·22) during weeks 53–102. In the vaccinated cohort, the incidence of type 2 diabetes was not markedly elevated in the first 4 weeks after COVID-19 without hospitalisation. Patterns of aHRs estimated within shorter time intervals during the first 16 weeks after COVID-19 were similar to those of the main analyses (appendix p 17). The aHRs for persistent type 2 diabetes after COVID-19 with hospitalisation and after COVID-19 without hospitalisation were consistent with those for all incident type 2 diabetes (table 3, appendix p 16). In each cohort, and after both COVID-19 with hospitalisation and COVID-19 without hospitalisation, a substantial proportion of type 2 diabetes diagnoses during weeks 1–4 were on the day of COVID-19 diagnosis (appendix pp 11, 18), and the aHRs were markedly higher on the day of COVID-19 diagnosis than during the rest of weeks 1–4 (appendix pp 11, 18).

The aHRs comparing the incidence of type 2 diabetes after COVID-19 with the incidence before or in the absence of COVID-19 were higher in older than in younger age groups during weeks 1–4. Differences in aHRs between age groups were small during subsequent time periods (appendix pp 12–13, 19). No marked differences were observed between aHRs for type 2 diabetes by sex or between ethnic groups. In the pre-vaccination cohort, the aHR for type 2 diabetes during weeks 1–4 after COVID-19 diagnosis was higher for people without than for people with obesity, and for people without than for people with prediabetes. In both the vaccinated and unvaccinated cohorts, aHRs for type 2 diabetes in people with a COVID-19 diagnosis before the cohort start date could not be estimated because there were too few (<50) incident type 2 diabetes events after a further COVID-19 diagnosis during the cohort follow-up.

Estimated excess risks of type 2 diabetes 6 months after COVID-19, standardised to the age and sex distribution of the pre-vaccination cohort, were 135 per 100 000 people diagnosed with COVID-19 in the pre-vaccination cohort, 58 in the vaccinated cohort, and 225 in the unvaccinated cohort (appendix p 20). In each cohort, absolute differences in risk were higher in people older than 60 years than in younger age groups. Little difference was observed in excess risk by sex, ethnicity, and presence or absence of prediabetes and obesity.

**Figure 2:**
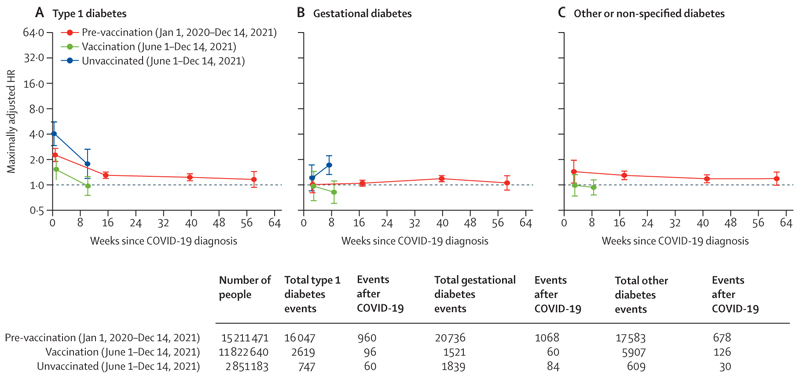
Maximally adjusted HRs comparing the incidence of type 1, gestational, and other diabetes events after COVID-19 with the incidence before or in the absence of COVID-19 in all cohorts Points are plotted at the median time of the outcome event within each follow-up period in each cohort. Error bars are 95% CI. HR=hazard ratio.

The aHRs comparing the incidence of type 1 diabetes after COVID-19 with the incidence before or in the absence of COVID-19 were higher during weeks 1–4 in all three cohorts, and higher in the pre-vaccination (2·27 [1·88–2·75]) and unvaccinated (4·06 [2·90–5·69]) cohorts than in the vaccinated cohort (1·53 [1·14–2·06]; table 3, figure 2). In the pre-vaccination cohort, the incidence of type 1 diabetes remained elevated during weeks 29–52 (aHR 1·23 [1·11–1·37]) but not during weeks 53–102 (1·16 [0·92–1·46]). In the pre-vaccination cohort, aHRs for type 1 diabetes incidence were markedly higher after COVID-19 with hospitalisation than for COVID-19 without hospitalisation. No consistent evidence of excess incidence of gestational diabetes after COVID-19 was observed in any group. The incidence of other or non-specified types of diabetes was also elevated after COVID-19, most markedly during the first year after hospitalisation with COVID-19 in the pre-vaccination cohort (figure 2).

## Discussion

In the cohort who were exposed to COVID-19 before vaccines were available, the incidence of type 2 diabetes was four times higher during the first 4 weeks after a diagnosis of COVID-19 than before or in the absence of COVID-19. Type 2 diabetes incidence remained elevated by 64% overall during the second year after diagnosis, was twice as high in people who were hospitalised with COVID-19, and was 11% higher in those not hospitalised with COVID-19. The majority of incident type 2 diabetes after COVID-19 was persistent. The increase in incidence of type 2 diabetes after COVID-19 was markedly attenuated in vaccinated compared with unvaccinated people (1·6 times higher vs 8·8 times higher during weeks 1–4 after COVID-19 diagnosis). The incidence of type 1 diabetes was elevated only during the first year after COVID-19 diagnosis, and the incidence of gestational diabetes did not seem to be elevated after COVID-19 diagnosis.

Increased incidence of diabetes (mostly type 2 diabetes) up to 1 year after COVID-19 has been reported in a 2023 systematic review.^20^ However, the largest previous study in the UK did not find an increase in diabetes incidence beyond 12 weeks after COVID-19.^5^ In that study, 20% of COVID-19 diagnoses were not laboratory-confirmed, potentially attenuating the association between COVID-19 and incident diabetes. Additionally, people with pre-existing cardiovascular disease were excluded, thereby removing a group that is susceptible to both COVID-19 and type 2 diabetes.

We found little variation in post-COVID-19 HRs for incident type 2 diabetes between subgroups defined by ethnicity, sex, and prediabetes or obesity. This finding implies that absolute differences in excess risk after COVID-19 are in people with a higher risk of developing type 2 diabetes, for example those belonging to specific ethnic groups and in people with prediabetes and obesity.

Around 60% of cases of incident type 2 diabetes persisted—defined as being on glucose-lowering medication or having an HbA_1c_ concentration consistent with type 2 diabetes at 4 months after diagnosis—with a similar proportion of persistent type 2 diabetes diagnoses observed in people after COVID-19 (57%) and before or in the absence of COVID-19 (62%). Using a similar definition of persistence, 56% of all cases of newly diagnosed type 2 diabetes persisted up to 1 year post-COVID-19 in a previous study.^21^ Additionally, 35% of newly diagnosed cases of prediabetes after COVID-19 persisted at 6 months.^21^ These previous studies were in patients who were hospitalised; we found slightly higher levels of persistence in people who were hospitalised (837 [61%] of 1382) than in those who were not hospitalised (2649 [56%] of 4731).

To our knowledge, no previous study has examined incident type 2 diabetes after COVID-19 diagnosis stratified by multiple periods of follow-up, and some excluded type 2 diabetes events that happened in the first 30 days after a COVID-19 diagnosis.^3^ Yet, the very high incidence of type 2 diabetes—including at the time of COVID-19 diagnosis in those hospitalised—should not be overlooked, given that the majority of these cases persist. Explanations for elevated incidence of type 2 diabetes around the time of COVID-19 diagnosis include routine tests unmasking undiagnosed diabetes or acute infection stress precipitating hyperglycaemia in those predisposed to diabetes. Incident diabetes occurring later after COVID-19 diagnosis could reflect direct damage to pancreatic β cells or systemic inflammatory responses in those who were not previously at risk.^22^ SARS-CoV-2 infection could be associated with a heightened risk of adverse metabolic states (eg, dyslipidaemia) and several non-communicable diseases,^23^ suggesting viral infection as a potential cause of chronic disease. The HR for incident type 2 diabetes between 5 weeks and 28 weeks after COVID-19 in the vaccinated cohort (1·20) was lower than that found for the pre-vaccination cohort (1·34).

Pre-vaccination, the incidence of type 1 diabetes was elevated 1–4 weeks after COVID-19, and this elevation persisted during weeks 29–52 both in patients who were hospitalised with COVID-19 and, to a lesser extent, in those who were not hospitalised with COVID-19; however, the elevation did not persist during the second year. This finding is consistent with both previous studies in adults (aged ≥18 years)^4^ and a meta-analysis of children and adolescents (aged ≤18 years),^24^ but is inconsistent with a study of children and young adults (aged ≤18 years) in Scotland, which observed an excess risk only in the month after COVID-19.^11^ Our findings of persistently elevated type 1 diabetes incidence (up to 1 year after infection), and the marked attenuation of associations between COVID-19 and type 1 diabetes in the vaccinated cohort, suggest that associations are not merely due to the higher likelihood of ascertaining COVID-19 around the time of diabetes diagnosis or vice versa.

We found no clear increase in the incidence of gestational diabetes after COVID-19, although there was some indication of small increases in the incidence of other forms of diabetes that persisted beyond 9 months. We are not aware of any previous studies examining gestational or non-specific diabetes subtypes.

A key strength is our sample size of 16 699 943 people, enabling comparisons by diabetes type, vaccination status, and time period after infection in many population subgroups, which—to our knowledge—no other study has done. Another strength is generalisability: the sample population was representative of the UK’s age, sex, and ethnicity distributions. The linkage of primary and secondary care data with national SARS-CoV-2 testing data maximised and made more precise the ascertainment of COVID-19 and other covariates, and enabled the study of hospitalised and non-hospitalised groups. All analyses accounted precisely for calendar time, so fluctuations in the incidence of COVID-19 and in the availability of health services will not have affected our findings.

The study has several limitations. Mild or asymptomatic cases of COVID-19 will have been underascertained before widespread testing was available, biasing associations towards the null. The ascertainment of prevalent, rather than incident, diabetes might have been more likely in those with COVID-19, especially as diabetes predisposes to severe COVID-19. We addressed this possibility with sensitivity analyses that excluded diabetes diagnoses made on the same day as COVID-19 diagnosis. Further, increased incidence of diabetes after COVID-19 was evident in the non-hospitalised population, who were less likely to have diabetes tests in the acute period. Conversely, under-ascertainment of diabetes could have occurred owing to the reduced use of primary care services throughout the pandemic and the cessation of normal screening activities, which would bias associations towards the null. The effect of different COVID-19 variants on subsequent diabetes diagnosis could not be directly accounted for because individual-level information was not available. To mitigate some of this effect, the cohorts were aligned to periods when specific variants were dominant in the population. Treatment of COVID-19 evolved and improved during the pandemic, which could have contributed to differences in HRs when comparing the pre-vaccination cohort with the vaccinated and unvaccinated cohorts, particularly after COVID-19 with hospitalisation. We cannot exclude the possibility that associations were driven in part by testing for SARS-CoV-2 being more likely in individuals at high risk of diabetes, although we controlled for an extensive range of comorbidities including prediabetes. Finally, bidirectional misclassification of diagnosis between type 2 and type 1 diabetes is likely to have occurred^17^ in those with acute hyperglycaemia in secondary care settings in this study and in the first year after diagnosis. Although our algorithm, validated against long-term clinical outcomes, might have gone some way to overcome this, we acknowledge some residual misclassification.^17^

Our findings have implications for the management and subsequent long-term consequences of the COVID-19 pandemic and potentially for future pandemics. England had around 2 million cases of COVID-19 during the pre-vaccination period and 18 million in the post-vaccination period.^25^ Accounting for non-vaccination, using our estimates of absolute numbers of people with incident type 2 diabetes by vaccination status and Office of National Statistics numbers of cases by hospitalisation, we estimate around 8700 additional new cases of type 2 diabetes in the 6 months after COVID-19 over the period of our study. This figure contrasts with an estimated 56 000 new cases of type 2 diabetes each year in England between 2015 and 2020.^26^ We acknowledge the assumptions and uncertainties associated with such calculations, including the effects of booster vaccination and waning vaccine effectiveness, the effects of changing variants on hospitalisation risk and disease severity, re-infections, and improvements in treatment. Although the estimate is markedly lower than a previous one,^27^ it still remains an alarmingly high number of new cases of type 2 diabetes, with substantial costs to individuals and society. Encouraging vaccination—which, in addition to reducing the immediate severity of COVID-19, reduces the immediate and longer-term risk of incident type 2 diabetes after COVID-19—is essential. Routine testing for diabetes after severe COVID-19, particularly in people at previous elevated risk of diabetes, and ensuring treatment and continued monitoring to identify those whose diabetes persists or resolves, should be considered. That COVID-19 appears to increase the incidence of type 1 diabetes^28^ and possibly type 2 diabetes^29,30^ to a greater extent than other respiratory infections adds weight to this recommendation. Our finding that the incidence of type 2 diabetes remains elevated up to 2 years after COVID-19 in the unvaccinated cohort emphasises the need to extend these analyses with longer follow-up.

## Supplementary Material

Supplementary appendix

## Figures and Tables

**Table 1:** Patient baseline characteristics

	Pre-vaccination cohort (Jan 1, 2020–Dec 14, 2021)			Vaccinated cohort (June 1–Dec 14, 2021)			Unvaccinated cohort (June 1–Dec 14, 2021)	
	n or n (%)	COVID-19 diagnoses		n or n (%)	COVID-19 diagnoses		n or n (%)	COVID-19 diagnoses
All	16 699 943	916 802		12 279 669	774 475		3 076 951	153 941
Sex								
Female	8 436 200 (50·5%)	497 312		6 474 117 (52·7%)	435 929		1 285 036 (41·8%)	81 864
Male	8 263 743 (49·5%)	419 490		5 805 552 (47·3%)	338 546		1 791 915 (58·2%)	72 077
Age, years								
18–29	3 159 485 (18·9%)	243 445		1 676 890 (13·7%)	90 196		948 183 (30·8%)	42 075
30–39	3 073 810 (18·4%)	195 819		1 837 906 (15·0%)	138 794		933 224 (30·3%)	52 769
40–49	2 847 856 (17·1%)	175 491		2 027 884 (16·5%)	218 155		587 009 (19·1%)	34 843
50–59	2 912 340 (17·4%)	161 341		2 400 275 (19·5%)	182 210		346 430 (11·3%)	16 781
60–69	2 135 491 (12·8%)	72 365		1 920 455 (15·6%)	87 056		164 159 (5·3%)	5081
70–79	1 643 645 (9·8%)	36 174		1 575 039 (12·8%)	41 629		67 347 (2·2%)	1524
80–89	754 494 (4·5%)	21 997		691 778 (5·6%)	13 002		23 749 (0·8%)	681
90+	172 822 (1·0%)	10 170		149 442 (1·2%)	3433		6850 (0·2%)	187
Ethnicity								
White	13 066 140 (78·2%)	693 760		10 129 789 (82·5%)	661 320		1 901 007 (61·8%)	117 381
Mixed	196 753 (1·2%)	13 058		112 473 (0·9%)	6752		75 896 (2·5%)	3871
South Asian	989 344 (5·9%)	98 342		625 968 (5·1%)	33 103		296 309 (9·6%)	9510
Black	350 019 (2·1%)	23 847		180 078 (1·5%)	8139		159 152 (5·2%)	7536
Other	385 931 (2·3%)	18 366		208 115 (1·7%)	9457		180 855 (5·9%)	4108
Missing	1 711 756 (10·3%)	69 429		1 023 246 (8·3%)	55 704		463 732 (15·1%)	11 535
Index of multiple deprivation quintile								
1: Most deprived	3 157 082 (18·9%)	218 822		1 923 914 (15·7%)	11 6392		906 684 (29·5%)	46 065
2	3 293 852 (19·7%)	197 386		2 266 904 (18·5%)	140 826		738 638 (24·0%)	36 457
3	3 616 435 (21·7%)	184 437		2 721 915 (22·2%)	167 077		619 531 (20·1%)	30 667
4	3 437 814 (20·6%)	168 319		2 718 197 (22·1%)	173 806		475 384 (15·4%)	23 549
5: Least deprived	3 194 760 (19·1%)	147 838		2 648 739 (21.6%)	176 374		336 714 (10·9%)	17 203
Region								
East	3 892 858 (23·3%)	208 302		2 902 290 (23·6%)	166 228		697 917 (22·7%)	35 407
East Midlands	2 905 097 (17·4%)	172 149		2 152 234 (17·5%)	142 458		501 275 (16·3%)	29 305
London	1 129 655 (6·8%)	61 278		637 415 (5·2%)	34 032		442 633 (14·4%)	11 448
North East	804 164 (4·8%)	55 477		587 530 (4·8%)	44 317		131 350 (4·3%)	7485
North West	1 464 107 (8·8%)	98 616		1 098 343 (8·9%)	77 364		205 749 (6·7%)	12 258
South East	1 137 304 (6·8%)	47 393		862 383 (7·0%)	52 025		188 551 (6·1%)	9367
South West	2 348 495 (14·1%)	72 653		1 917 734 (15·6%)	117 750		306 317 (10%)	18 279
West Midlands	665 742 (4·0%)	49 566		424 247 (3·5%)	26 171		167 800 (5·5%)	8006
Yorkshire/Humber	2 352 521 (14·1%)	151 368		1 697 493 (13·8%)	114 130		435 359 (14·1%)	22 386
Smoking status								
Never smoker	7 791 912 (46·7%)	464 376		5 843 241 (47·6%)	383 295		1 277 902 (41·5%)	61 826
Ever smoker	5 244 708 (31·4%)	282 893		4 286 778 (34·9%)	290 095		598 989 (19·5%)	44 311
Current smoker	2 908 101 (17·4%)	127 609		1 733 339 (14·1%)	84 980		836 223 (27·2%)	38 814
Missing	755 222 (4·5%)	41 924		416 311 (3·4%)	16 105		363 837 (11·8%)	8990
Care home resident	24 814 (0·2%)	70 697 (0·4%)		11 714	44 945 (0·4%)		2181	2606 (0·1%)

**Table 2:** Number of diabetes events in each cohort, with person-years of follow-up, by COVID-19 severity

	Pre-vaccination cohort (n=16 674 867) median follow-up 714 (IQR 714-714) days			Vaccinated cohort (n=12 279 669) median follow-up 190 (IQR 147-197) days			Unvaccinated cohort (n=3 076 953) median follow-up 126 (IQR 106-172) days	
	Event (person-years)	Incidence rate*		Event/person-years	Incidence rate*		Event/person-years	Incidence rate*
**All participants**								
Type 2 diabetes								
No COVID-19	139 209 (31 273 857)	445		33 423 (5 499 661)	608		2535 (1 143 275)	222
Hospitalised COVID-19	1509 (33 944)	4446		171 (1718)	9953		153 (1526)	10 029
Non-hospitalised COVID-19	4815 (891 523)	540		771 (141 203)	546		93 (25 817)	360
Type 1 diabetes								
No COVID-19	15 087 (31 385 955)	48		2523 (5 507 811)	46		687 (1 143 784)	60
Hospitalised COVID-19	63 (35 505)	177		3 (1757)	171		21 (1554)	1351
Non-hospitalised COVID-19	897 (896 274)	100		93 (141 453)	66		39 (25 834)	151
Gestational diabetes								
No COVID-19	19 671 (15 807 408)	124		1461 (2 956 484)	49		1755 (475 611)	369
Hospitalised COVID-19	27 (15 731)	172		3 (832)	360		3 (708)	424
Non-hospitalised COVID-19	1041 (488 190)	213		57 (79 182)	72		81 (13 249)	611
Other or non-specified diabetes								
No COVID-19	16 905 (31 388 172)	54		5781 (5 507 103)	105		579 (1 143 781)	51
Hospitalised COVID-19	93 (35 499)	262		9 (1755)	513		9 (1556)	578
Non-hospitalised COVID-19	585 (896 842)	65		117 (141 449)	83		21 (25 841)	81
**Restricted to participants with at least 4 months follow-up**								
Type 2 diabetes								
No COVID-19	139 209 (31 258 556)	445		· ·	· ·		· ·	· ·
Hospitalised COVID-19	1383 (33 382)	4143		· ·	· ·		· ·	· ·
Non-hospitalised COVID-19	4731 (890 542)	531		· ·	· ·		· ·	· ·
Persistent type 2 diabetes				· ·	· ·		· ·	· ·
No COVID-19	86 031 (31 291 469)	275		· ·	· ·		· ·	· ·
Hospitalised COVID-19	837 (33 868)	2471		· ·	· ·		· ·	· ·
Non-hospitalised COVID-19	2649 (891 982)	297		· ·	· ·		· ·	· ·

* Incidence rates are per 100 000 person-years.

**Table 3:** aHRs comparing the incidence of diabetes events in the weeks after COVID-19 with the incidence before or without COVID-19, in all cohorts, overall and according to COVID-19 severity

	Pre-vaccination cohort aHR (95% CI)		Vaccinated cohort aHR (95% CI)	Unvaccinated cohort aHR (95% CI)
	All	Persistent (treated for ≥4 months)		
**Type 2 diabetes**				
All COVID-19; age, sex, and region-adjusted				
1–4 weeks	5·27 (4·98–5·58)	3·51 (3·25–3·79)	1·63 (1·47–1·80)	10·26 (8·77–12·00)
5–28 weeks	1·67 (1·61–1·74)	1·63 (1·56–1·71)	1·21 (1·11–1·31)	2·42 (1·92–3·04)
29–52 weeks	1·50 (1·44–1·57)	1·57 (1·46–1·69)	· ·	· ·
53–102 weeks	1·64 (1·51–1·78)	1·94 (1·64–2·29)	· ·	· ·
All COVID-19; maximally adjusted				
1–4 weeks	4·30 (4·06–4·55)	3·08 (2·85–3·33)	1·66 (1·50–1·84)	8·76 (7·49–10·25)
5–28 weeks	1·38 (1·32–1·43)	1·43 (1·37–1·50)	1·20 (1·10–1·31)	1·99 (1·58–2·50)
29–52 weeks	1·22 (1·17–1·28)	1·33 (1·23–1·43)	· ·	· ·
53–102 weeks	1·24 (1·14–1·35)	1·53 (1·30–1·81)	· ·	· ·
Hospitalised COVID-19; maximally adjusted				
1–4 weeks	28·26 (26·19–30·49)	24·16 (21·81–26·77)	20·99 (17·77–24·79)	54·54 (44.99–66·13)
5–28 weeks	2·75 (2·49–3·03)	3·24 (2·89–3·63)	3·15 (2·28–4·35)	6·04 (4·13–8·82)
29–52 weeks	1·84 (1·62–2·09)	2·40 (1·99–2·90)	· ·	· ·
53–102 weeks	2·04 (1·72–2·42)	2·30 (1·70–3·12)	· ·	· ·
Non·hospitalised COVID·19; maximally adjusted				
1–4 weeks	1·95 (1·78–2·13)	1·40 (1·25–1·58)	1·11 (0·98–1·25)	2·63 (1·96–3·52)
5–28 weeks	1·26 (1·20–1·31)	1·31 (1·24–1·37)	1·13 (1·04–1·24)	1·42 (1·06–1·89)
29–52 weeks	1·17 (1·11–1·23)	1·23 (1·13–1·33)	· ·	· ·
53–102 weeks	1·11 (1·01–1·22)	1·35 (1·11–1·65)	· ·	· ·
**Type 1 diabetes**				
All COVID-19; age sex, and region-adjusted				
1–4 weeks	2·44 (2·02–2·96)	· ·	1·60 (1·19–2·15)	4·91 (3·51–6·87)
5–28 weeks	1·41 (1·28–1·56)	· ·	1·04 (0·80–1·37)	2·25 (1·49–3·39)
29–52 weeks	1·34 (1·21–1·50)	· ·	· ·	· ·
53–102 weeks	1·31 (1·04·1–65)	· ·	· ·	· ·
All COVID-19; maximally adjusted				
1–4 weeks	2·27 (1·88–2·75)	· ·	1·53 (1·14–2·06)	4·06 (2·90–5·69)
5–28 weeks	1·30 (1·18–1·44)	· ·	0·97 (0·74–1·27)	1·78 (1·18–2·69)
29–52 weeks	1·23 (1·11–1·37)	· ·	· ·	· ·
53–102 weeks	1·16 (0·92–1·46)	· ·	· ·	· ·
Hospitalised COVID-19; maximally adjusted				
1–4 weeks	51·31 (35·74–73·65)	· ·	*	*
5–28 weeks	5·61 (3·65–8·63)	· ·	*	*
29–52 weeks	1·79 (0·80–4·00)	· ·	· ·	· ·
53–102 weeks	2·63 (0·98–7·04)	· ·	· ·	· ·
Non-hospitalised COVID-19; maximally adjusted				
1–4 weeks	1·65 (1·33–2·06)	· ·	1·39 (1·02–1·90)	*
5–28 weeks	1·24 (1·12–1·38)	· ·	0·95 (0·73–1·25)	*
29–52 weeks	1·21 (1·09–1·35)	· ·	· ·	· ·
53–102 weeks	1·10 (0·87–1·39)	· ·	· ·	· ·
**Gestational diabetes**				
All COVID-19; age, sex, and region-adjusted				
1–4 weeks	1·12 (0·88–1·41)	· ·	0·98 (0·65–1·48)	1·38 (0·96–2·00)
5–28 weeks	1·16 (1·06–1·28)	· ·	0·83 (0·61–1·15)	2·01 (1·53–2·63)
29–52 weeks	1·33 (1·21–1·46)	· ·	· ·	· ·
53–102 weeks	1·25 (1·02–1·55)	· ·	· ·	· ·
All COVID-19; maximally adjusted				
1–4 weeks	1·00 (0·79–1·26)	· ·	0·96 (0·63–1·45)	1·20 (0·83–1·73)
5–28 weeks	1·04 (0·94–1·14)	· ·	0·81 (0·59–1·12)	1·70 (1·30–2·23)
29–52 weeks	1·17 (1·07–1·29)	· ·	· ·	· ·
53–102 weeks	1·04 (0·85–1·29)	· ·	· ·	· ·
Hospitalised COVID-19; maximally adjusted				
1–4 weeks	*	· ·	*	*
5–28 weeks	*	· ·	*	*
29–52 weeks	*	· ·	· ·	· ·
53–102 weeks	*	· ·	· ·	· ·
Non-hospitalised COVID-19; maximally adjusted				
1–4 weeks	0·88 (0·68–1·13)	· ·	0·91 (0·60–1·39)	1·15 (0·79–1·68)
5–28 weeks	1·02 (0·93–1·13)	· ·	0·79 (0·57–1·09)	1·64 (1·24–2·17)
29–52 weeks	1·18 (1·07–1·30)	· ·	· ·	· ·
53–102 weeks	1·05 (0·85–1·30)	· ·	· ·	· ·
**Other or non-specified diabetes**				
All COVID-19; age, sex, and region-adjusted				
1–4 weeks	1·78 (1·29–2·46)	· ·	0·99 (0·74–1·34)	*
5–28 weeks	1·61 (1·42–1·83)	· ·	0·96 (0·78–1·19)	*
29–52 weeks	1·47 (1·31–1·66)	· ·	· ·	· ·
53–102 weeks	1·63 (1·35–1·97)	· ·	· ·	· ·
All COVID-19; maximally adjusted				
1–4 weeks	1·45 (1·05–2·00)	· ·	1·00 (0·74–1·36)	*
5–28 weeks	1·32 (1·16–1·49)	· ·	0·95 (0·77–1·18)	*
29–52 weeks	1·20 (1·06–1·35)	· ·	· ·	· ·
53–102 weeks	1·20 (0·99–1·46)	· ·	· ·	· ·
Hospitalised COVID-19; maximally adjusted				
1–4 weeks	4·34 (2·38–7·89)	· ·	*	*
5–28 weeks	2·28 (1·61–3·22)	· ·	*	*
29–52 weeks	2·22 (1·61–3·05)	· ·	· ·	· ·
53–102 weeks	1·14 (0·66–1·97)	· ·	· ·	· ·
Non-hospitalised COVID-19; maximally adjusted				
1–4 weeks	1·23 (0·86–1·78)	· ·	0·94 (0·69–1·29)	*
5–28 weeks	1·26 (1·10–1·44)	· ·	0·91 (0·73–1·14)	*
29–52 weeks	1·14 (1·00–1·29)	· ·	· ·	· ·
53–102 weeks	1·24 (1·01–1·52)	· ·	· ·	· ·

aHR=adjusted hazard ratio.

* Insufficient events for estimation.

## Data Availability

Access to the underlying identifiable and potentially re-identifiable pseudonymised electronic health record data is tightly governed by various legislative and regulatory frameworks, and restricted by best practice. The data in OpenSAFELY is drawn from general practice data across England, where TPP is the data processor. TPP developers initiate an automated process to create pseudonymised records in the core OpenSAFELY database, which are copies of key structured data tables in the identifiable records. These pseudonymised records are linked onto key external data resources that have also been pseudonymised via SHA-512 one-way hashing of NHS numbers using a shared salt. Bennett Institute for Applied Data Science developers and principle investigators holding contracts with NHS England have access to the OpenSAFELY pseudonymised data tables as needed to develop the OpenSAFELY tools. These tools in turn enable researchers with OpenSAFELY data access agreements to write and execute code for data management and data analysis without direct access to the underlying raw pseudonymised patient data, and to review the outputs of this code. All code for the full data management pipeline—from raw data to completed results for this analysis—and for the OpenSAFELY platform as a whole is available for review at https://github.com/OpenSAFELY. Individual-level data for this project are not available for sharing. The code to write and execute code for data management and data analysis is available on GitHub. Accredited researchers can apply to OpenSAFELY to execute any of the publicly available code on the individual-level data. Data dictionaries and metadata are available in the OpenSAFELY documentation (https://docs.opensafely.org/). The code to create the dummy data used to write data curation and data analysis code is available on the GitHub repository (Post-COVID diabetes events in the era of delta among the fully vaccinated and the electively unvaccinated; https://github.com/OpenSAFELY/post-covid-diabetes). All released outputs used in this manuscript are available on the OpenSAFELY job server (https://jobs.opensafely.org/investigating-events-following-sars-cov-2-infection/post-covid-diabetes/outputs/).

## Research in context

### Evidence before this study

We searched PubMed for population-based observational studies published between Dec 1, 2019, and July 12, 2023, examining associations between SARS-CoV-2 infection or COVID-19 diagnosis (search string: SARS-CoV-2 or COVID* or coronavirus*) and subsequent incident diabetes (search term: diabetes). Of 19 relevant studies, eight had a composite outcome of diabetes types, six stratified by diabetes type, and five pertained to type 1 diabetes only. We did not identify any studies relating to gestational or non-specific types of diabetes. 11 studies were from the USA, three from the UK, two from Germany, one from Canada, one from Denmark, and one from South Korea. Most studies described cumulative relative risks of incident diabetes 1–2 years after-SARS-CoV-2 infection of 1·2–2·6 (for infection *vs* no infection), although four studies found no associations with type 1 diabetes after the post-acute period. All studies lacked the power to compare the relative risk of diabetes by type, COVID-19 severity, and COVID-19 vaccination status in population subgroups. One study examined relative risks by vaccination status using a composite outcome of diabetes and hyperlipidaemia in a cohort consisting mainly of White men. Two studies of type 1 diabetes found no evidence of increased risk beyond 30 days after COVID-19 diagnosis, whereas two reported increased risks at 6 months. Two studies of type 2 diabetes examined relative risks by time period post-infection: one study of US insurance claims reported a persistent association 6 months after infection, whereas a large UK population-based study, using primary care data only, reported no associations after 12 weeks. No large studies have investigated whether all patients diagnosed with diabetes after a COVID-19 diagnosis continued to receive long-term (>4 months) treatment in the subsequent months, which is key to elucidating the role of stress-induced or steroid-induced hyperglycaemia.

### Added value of this study

This study—which, to our knowledge, is the largest to date to address the association between COVID-19 and different subtypes of diabetes and the role of COVID-19 vaccination—analysed linked primary and secondary care health records with SARS-CoV-2 testing and COVID-19 vaccination data for 16 million people living in England. This analysis enabled us to compare the increase in incident diabetes diagnosis after COVID-19 diagnosis by diabetes type, COVID-19 severity, and COVID-19 vaccination status, overall and in population subgroups. Notably, excess diabetes incidence by time period since infection could also be quantified. Because health care in the UK is universal and free at the point of delivery, almost the entire population is registered with primary care. The findings are therefore likely to be generalisable to the UK and internationally. We found that, before the availability of COVID-19 vaccination, a COVID-19 diagnosis (*vs* no diagnosis) was associated with increased incidence of type 2 diabetes, which remained elevated by approximately 30% beyond 1 year after diagnosis. Although still present (with around 30% excess incidence at 8 weeks), these associations were substantially attenuated in vaccinated compared with unvaccinated people. Excess incidence was greater in people who were hospitalised with COVID-19 than those who were not hospitalised after diagnosis. The incidence of type 1 diabetes was elevated up to, but not beyond, 1 year after COVID-19 diagnosis. Around 60% of people diagnosed with incident type 2 diabetes after COVID-19 still had evidence of diabetes 4 months after infection.

### Implications of all the available evidence

Diagnosis of incident type 2 diabetes increases by 30–50% after COVID-19, but we report the new finding of elevated incidence beyond 1 year after COVID-19 diagnosis. The increased incidence of type 1 diabetes did not seem to persist beyond 1 year, which could explain the disparities between previous studies. In a general-population dataset we show that COVID-19 vaccination reduces, but does not entirely ameliorate, excess diabetes incidence after COVID-19. This finding supports a policy of universal vaccination and suggests that other public health activities—such as enhanced diabetes screening after severe COVID-19—might be warranted, particularly in unvaccinated people.
